# Development and validation of an efficient nomogram for risk assessment of norovirus infection in pediatric patients

**DOI:** 10.1007/s10096-022-04510-8

**Published:** 2022-10-25

**Authors:** Taojun He, Xiaohua Chen, Yilin Deng, Bin Li, Hongmei Wang, Qinjin Wang, Aixia Zhai, Liang Shi, Ying Chen, Chao Wu

**Affiliations:** 1 Department of Laboratory Medicine, The Eighth Affiliated Hospital of Sun Yat-Sen University, Shenzhen, 518033 Guangdong Province China; 2 Department of Digestive Endoscopy Center, The Eighth Affiliated Hospital of Sun Yat-Sen University, Shenzhen, 518033 Guangdong Province China; 3 Department of Infectious Diseases, Shenzhen Children’s Hospital, Shenzhen, 518000 Guangdong Province China

**Keywords:** Norovirus infection, Risk assessment, Prediction model, Accuracy, Clinical decision-making

## Abstract

This study aimed to establish a predictive model and nomogram based on routine laboratory blood indicators and clinical symptoms, subsequently providing a rapid risk assessment of norovirus (NoV) infection in children. This retrospective study enrolled 307 pediatric patients with symptoms of acute gastroenteritis and detected NoV using real-time quantitative polymerase chain reaction. Significant indicators selected by multivariate logistic regression, including routine blood tests and consultation symptoms, were used to develop the nomogram. We divided the sample into training and internal validation sets and performed external validation of the final model. Furthermore, we evaluated the clinical performance using the Akaike information criterion (AIC), area under the curve (AUC), calibration curve, decision curve analysis (DCA), sensitivity, specificity, concordance rate, positive predictive value, and negative predictive value. Overall, 153 cases were NoV-PCR-positive, and 154 were negative. The multivariate logistic regression included five predictors of NoV infection, including symptoms of vomiting, upper respiratory tract infection, and indicators of white blood cells, lymphocyte absolute counts, and platelet counts. The nomogram showed a significant predictive value with overall internal set diagnosis, with an AUC of 0.827 (95% confidence interval (CI): 0.785–0.868), and 0.812 (95% *CI*: 0.755–0.869) with 0.799 (95% *CI*: 0.705–0.894) in the training and internal validation sets, respectively. Nevertheless, the AUC in the external validation set was higher (0.915; 95% *CI*: 0.862–0.968). This nomogram is a useful tool for risk assessment for NoV infection. Moreover, the evaluated indicators are accessible, substantially reducing the time for laboratory testing.

**Supplementary Information:**

The online version contains supplementary material available at 10.1007/s10096-022-04510-8.

## Introduction

Norovirus (NoV) infection can cause severe gastrointestinal disease. Since the 1990s, NoV has caused at least four global outbreaks, affecting nearly 50–70% of the population across six regions [1–3]. Current data show that NoV is the main pathogen that causes acute gastroenteritis in children, typically infecting those younger than 5 years. Moreover, NoV infection can lead to a substantial increase in the prevalence and hospitalization rates of susceptible patients and even lead to the death of infants, older adults, and patients with immunodeficiency [4–8]. Therefore, rapid and accurate identification of pathogens is critical for treating patients, preventing and controlling nosocomial infections, and preventing community outbreaks.

Screening for NoV infection is not routinely performed in all hospitals. The main symptoms of NoV infection are vomiting, abdominal pain, and diarrhea, which can be easily confused with other gastrointestinal diseases [9, 10]. This undoubtedly causes difficulties in the differential diagnosis of NoV infections. Currently, nucleic acid-based real-time quantitative polymerase chain reaction (PCR) technology is the gold standard for NoV laboratory diagnosis, with a turnaround time of almost 5 h. However, genotype diversity and rapid evolution induced target failure [11–13], and thus the clinical diagnosis and treatment oftentimes will not be efficiently implemented.

Therefore, comprehensive and accurate risk assessment models for the diagnosis of NoV infection are essential for rapid diagnosis and treatment, subsequently preventing and controlling disease outbreaks. The nomogram is a statistical instrument that accounts for numerous variables to predict the outcome of an individual patient, and it is routinely used to assist in decision-making in cancer, trauma, neurocritical care, and other specialties [14–16].

The primary purpose of this study was to develop a risk assessment model using multivariate logistic regression based on a combination of routine laboratory blood indicators and clinical symptoms to diagnose NoV infections. In addition, we improved the evaluative performance and achieved higher accuracy by comparing multiple models with internal and external validation. Ultimately, the benefit of using the model was to save the patient from worse outcomes, shorten hospital stays, reduce economic burden, and control NoV transmission effectively, thus preventing new outbreaks of NoV infection.

## Materials and methods

### Patient selection and data collection

**Fig. 1:**
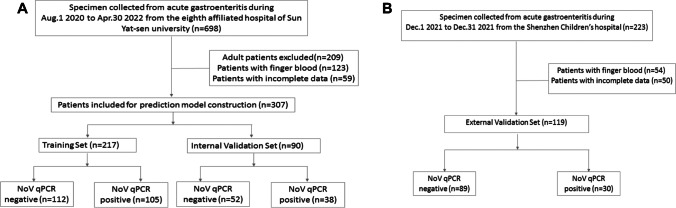
Flowchart of participant selection. **A** The Eighth Affiliated Hospital of Sun Yat-sen University set and **B** Shenzhen Children’s Hospital set

We collected 698 fecal specimens between August 1, 2020 and April 30, 2022 from the eighth affiliated hospital of Sun Yat-sen University that admitted patients with acute gastroenteritis (no. 2020–058-01). Moreover, 223 stool samples were collected from the Shenzhen Children’s Hospital from December 1 to December 31, 2021 (no. 201801504). Stool samples from patients with NoV infection are usually watery or loose, with no mucus or blood, and leukocytes are typically absent. The evaluation of the consistency of the stool using the Bristol scale has been considered. Patients aged between 3 months and 16 years with clinical suspicion of acute viral gastroenteritis were included. Meanwhile, patients with the following criteria were excluded: < 3 months old, toxic appearance or shock, suspected bacterial colitis, blood in stool, persistent localized abdominal pain or signs of obstruction, and other major comorbid medical conditions. First, a qPCR test was performed on all the specimens. We divided the samples into NoV-negative and NoV-positive groups based on qPCR results. Next, the eligible laboratory data and relevant information derived from the electronic medical records were used to establish a database for subsequent analysis (Fig. 1). The basic demographic characteristics of each cohort are shown in Table S1.

The database includes the demographic characteristics and 26 blood routine examination results and immune cell indicators, such as white blood cell (WBC) and absolute counts (neutrophils (NC), lymphocytes (LC), monocytes (MONO), eosinophils (EO), basophils (BO)); red blood cell (RBC) absolute counts and related parameters (hemoglobin (HGB), mean corpuscular volume (MCV), mean corpuscular hemoglobin (MCH), mean corpuscular hemoglobin concentration (MCHC), hematocrit (HCT), red cell volume distribution width (RDW-CV), and standard deviation of red blood cell distribution width (RDW-SD)); platelet absolute counts (PLT) and related parameters (mean platelet volume (MPV), platelet count (PCT), platelet distribution width (PDW), platelet-larger cell ratio (P-LCR)); and C-reactive protein (CRP). Moreover, the percentage of T cells (CD3 + , CD4 + , CD8 +) in whole blood and the ratio of CD4 + /CD8 + T cells were determined by flow cell count analysis.

In addition to collecting data from the laboratory information system (LIS), we collected information on the patient’s self-reported symptoms during admission and subsequent corresponding treatment under the physician’s order sheet through electronic medical records with checklists. Clinical symptoms included gastrointestinal discomfort such as diarrhea, vomiting, abdominal pain, and dehydration, as well as systemic symptoms such as fever, convulsions, and accompanying upper respiratory infection. Respiratory tract infections consist of bacterial and viral infections, typically occurring in the winter and spring. Co-infection of respiratory pathogens and NoV tends to overlap with the high season of NoV infection. These variables were sorted into categorical variables. The corresponding therapies are usually symptomatic and supportive treatment, single antibiotic therapy, more than two types of antibiotic therapy, and fluid replacement therapy.

### Real-time polymerase chain reaction testing for NoV infection

A total of 921 specimens were collected from the two hospitals. Following our criteria, 426 patient specimens were included in our final analysis. All fecal samples obtained from eligible patients were tested by PCR fluorescent probe detection using the NoV nucleic acid detection kit (Hubei Langde Medical Technology Co., Ltd., Hubei, CHN). Based on the qPCR results, we detected 153 positive and 154 negative samples at the eighth affiliated hospital of Sun Yat-sen University, and 30 positive and 89 negative samples in Shenzhen Children’s Hospital (Fig. 1).

### Statistical analyses

Univariate analyses were performed to identify independent predictors for the multivariate analysis. Pearson’s *χ*^2^ test or Fisher’s exact test was applied for univariate analysis of categorical variables. The continuous variable of laboratory results was presented as the mean with standard deviation. Differences between the NoV qPCR positive and negative groups were examined using a Student’s *t*-test. Statistically significant variables in the univariate analysis and previously known predictors were selected for inclusion in the multivariate logistic regression model.

### Construction of NoV infection risk assessment model

Based on routine laboratory blood test results combining clinical symptoms and treatments, we obtained several multivariate indicators to assess the risk of NoV infection. We developed a risk assessment model for 307 samples, and then randomly allocated 70% and 30% of the enrolled eligible patients to the training (*n* = 217) and internal validation set (*n* = 90), respectively. Subsequently, we collected another 119 samples to apply the external validation set. The nomogram was constructed based on the results of the multivariable analysis. Moreover, the regression coefficients were considered the weights of the variables in the risk evaluation model [14–16]. We built two models (models 1 and 2) by including different variables and finally selected model 2 with a smaller Akaike information criterion (AIC) to draw the nomogram. This model was applied to rapidly assess the possibility of NoV infection in clinical practice.

All statistical analyses, including the development and validation of logistic regression, ROC, and nomogram, were performed using SPSS Statistics version 22.0 (IBM Corp, New York, NY, USA) and STATE/SE 15.0 (V.15.0; Stata, College Station, TX, USA). All statistical tests were two tailed. A *p*-value < 0.05 was considered statistically significant.

## Results

### Patient characteristics

The study sample comprised 426 children, and the majority of whom (*n* = 264; 62%) were male. The mean patient age was approximately 3 years. No statistically significant differences were observed in the demographic characteristics between the NoV infection and non-infection groups (Table S1). The two groups with offset-free samples were thus considered comparable in subsequent data analysis.

### Univariate analysis of blood routine indicators, clinical manifestation, and therapies

The univariate logistic regression analysis of the two groups found that the index values of WBC (11.37 ± 5.73 vs. 8.74 ± 3.28), LC (4.01 ± 2.24 vs. 3.29 ± 1.69), NC (6.31 ± 5.45 vs. 4.57 ± 2.98), and PLT (372.51 ± 105.55 vs. 270.38 ± 75.74) in the NoV infection group were significantly higher than those in the non-infection group (*p* < 0.05, Table 1). Although other indicators were not statistically significant in the univariate analysis, it is worth noting that MONO and PCT levels were significantly higher in the positive compared to the negative group. Meanwhile, RBC and related indicators were nearly coincidental. We also calculated the percentages of CD3 + , CD4 + , and CD8 + T lymphocytes using flow cytometry. The comparison between the two groups revealed that the percentage of CD3 + and CD8 + T cells in the negative group was slightly higher than that in the positive group; however, the difference was not significant.

**Table 1:** Univariate analysis of NoV infection associations with the laboratory blood routine test variables in training set

Lab blood variable	NoV qPCR negative group ($\overline{x }$ ± s)	NoV qPCR positive group ($\overline{x }$ ± s)	*p*–value	*OR* (95% *CI*)
WBC (*10^9^/L)	8.74 ± 3.28	11.37 ± 5.73	** < 0.001**	1.143 (1.066–1.225)
NC (*10^9^/L)	4.57 ± 2.98	6.31 ± 5.45	**0.006**	1.102 (1.029–1.18)
LC (*10^9^/L)	3.29 ± 1.69	4.01 ± 2.24	**0.01**	1.211 (1.046–1.401)
MONO (*10^9^/L)	0.76 ± 0.44	0.87 ± 0.52	0.097	1.633 (0.916–2.913)
EO (*10^9^/L)	0.1 ± 0.18	0.15 ± 0.23	0.089	3.693 (0.819–16.655)
BO (*10^9^/L)	0.03 ± 0.02	0.03 ± 0.03	0.772	3.397 (0.001–13,424.133)
RBC (*10^12^/L)	4.51 ± 0.41	4.56 ± 0.5	0.395	1.291 (0.717–2.326)
HGB (g/L)	118.04 ± 10.78	117.58 ± 12.91	0.776	0.997 (0.975–1.019)
MCV (fl)	79.63 ± 5.91	78.59 ± 6.9	0.239	0.975 (0.935–1.017)
MCH (pg)	26.3 ± 2.27	25.96 ± 2.8	0.326	0.948 (0.853–1.054)
MCHC (g/L)	330.15 ± 13.62	329.81 ± 11.98	0.848	0.998 (0.977–1.019)
HCT	0.25 ± 0.16	0.26 ± 0.16	0.923	1.001 (0.985–1.017)
RDW–CV (%)	13.28 ± 1.75	13.68 ± 2.22	0.143	1.115 (0.964–1.291)
RDW–SD (fl)	38.32 ± 3.41	38.74 ± 4.7	0.455	1.025 (0.96–1.096)
PLT (*10^9^/L)	270.38 ± 75.74	372.51 ± 105.55	** < 0.001**	1.013 (1.009–1.017)
MPV (fl)	9.34 ± 1.11	9.39 ± 1.1	0.725	1.045 (0.819–1.333)
PCT (%)	0.25 ± 0.07	0.35 ± 0.1	0.359	1.098 (0.899–1.342)
PDW (%)	11.36 ± 2.81	11.18 ± 2.4	0.624	0.975 (0.879–1.08)
P–LCR (%)	20.51 ± 7.04	20.85 ± 7.35	0.730	1.007 (0.969–1.045)
CRP (mg/L)	18.45 ± 23.99	18.01 ± 32.53	0.917	0.999 (0.989–1.01)
Immature N (*10^9^/L)	0.05 ± 0.08	0.04 ± 0.07	0.649	0.13 (0.687–0.171)
Immature N (%)	0.24 ± 0.22	0.22 ± 0.18	0.809	0.658 (0.022–19.704)
CD3 + T (%)	67.6 ± 10.08	62.44 ± 9.63	0.107	0.946 (0.883–1.012)
CD4 + T (%)	36.13 ± 11.36	37.85 ± 8.65	0.562	1.02 (0.955–1.089)
CD8 + T (%)	26.55 ± 14.56	20.03 ± 6.84	0.091	0.931 (0.858–1.011)
CD4 + /CD8 +	1.77 ± 1.04	2.07 ± 0.79	0.270	1.543 (0.714–3.334)

The significance represented in bold is a statistically significant difference in comparison between the two groups

In terms of indicators of symptoms and corresponding treatments, we found that accompanying symptoms such as vomiting (odds ratio (OR): 0.232, 95% confidence interval (CI): 0.113–0.476, *p* < 0.001), diarrhea (*OR*: 2.442, 95% *CI*: 1.09–5.469, *p* = *0.03*), and URI (*OR*: 0.504, 95% *CI*: 0.288–0.883, *p* = 0.017) were significantly associated with NoV infection (Table 2).

**Table 2:** Univariate analysis of NoV infection associations with specific clinical symptoms in training set

Clinical symptoms	NoV qPCR negative group (*N* = 110)	NoV qPCR positive group (*N* = 107)	*p*–value	*OR* (95% *CI*)
Vomiting	12	37	** < 0.001**	0.232 (0.113–0.476)
Diarrhea	100	86	**0.03**	2.442 (1.09–5.469)
Abdominal pain	13	6	0.113	2.256 (0.824–6.173)
Fever	32	29	0.745	1.103 (0.61–1.996)
Cough	10	19	0.065	0.463 (0.204–1.049)
Convulsion	6	6	0.961	0.971 (0.303–3.111)
Dehydration	12	4	0.053	3.153 (0.984–10.107)
Upper respiratory infection	32	48	**0.017**	0.504 (0.288–0.883)
Community related	4	9	0.149	0.411 (0.123–1.377)

The significance represented in bold is a statistically significant difference in comparison between the two groups

These significant symptom indicators were then selected for multivariate analysis, and a risk assessment and prediction model were constructed. In addition, we found that the NoV qPCR-positive group had significantly more clinical symptoms and treatment methods adopted by doctors than the negative group in total samples (Table 3).

**Table 3:** Analysisof NoV infection associations with different degrees of symptoms and therapies in total samples

Symptom/therapy	NoV qPCR negative group (*N* = 243)	NoV qPCR positive group (*N* = 183)	*χ*^2^ value	*p*–value
Symptom				
Less than two symptoms	204	124	22.979	**0.001**
Three types of symptoms	32	34		
More than four symptoms	7	25		
Therapy				
Supportive treatment	75	35	45.460	**0.001**
Two types of treatment	131	66		
More than three treatments	37	82		

symptoms — vomiting, diarrhea, abdominal pain, fever, cough, convulsion, dehydration, upper respiratory infection, community related; therapy — symptomatic and supportive treatment, single antibiotics therapy, more than two types of antibiotics therapy, fluid replacement therapy

The significance represented in bold is a statistically significant difference in comparison between the two groups

### Multivariate analysis and construction of nomogram risk prediction model for NoV infection

**Fig. 2:**
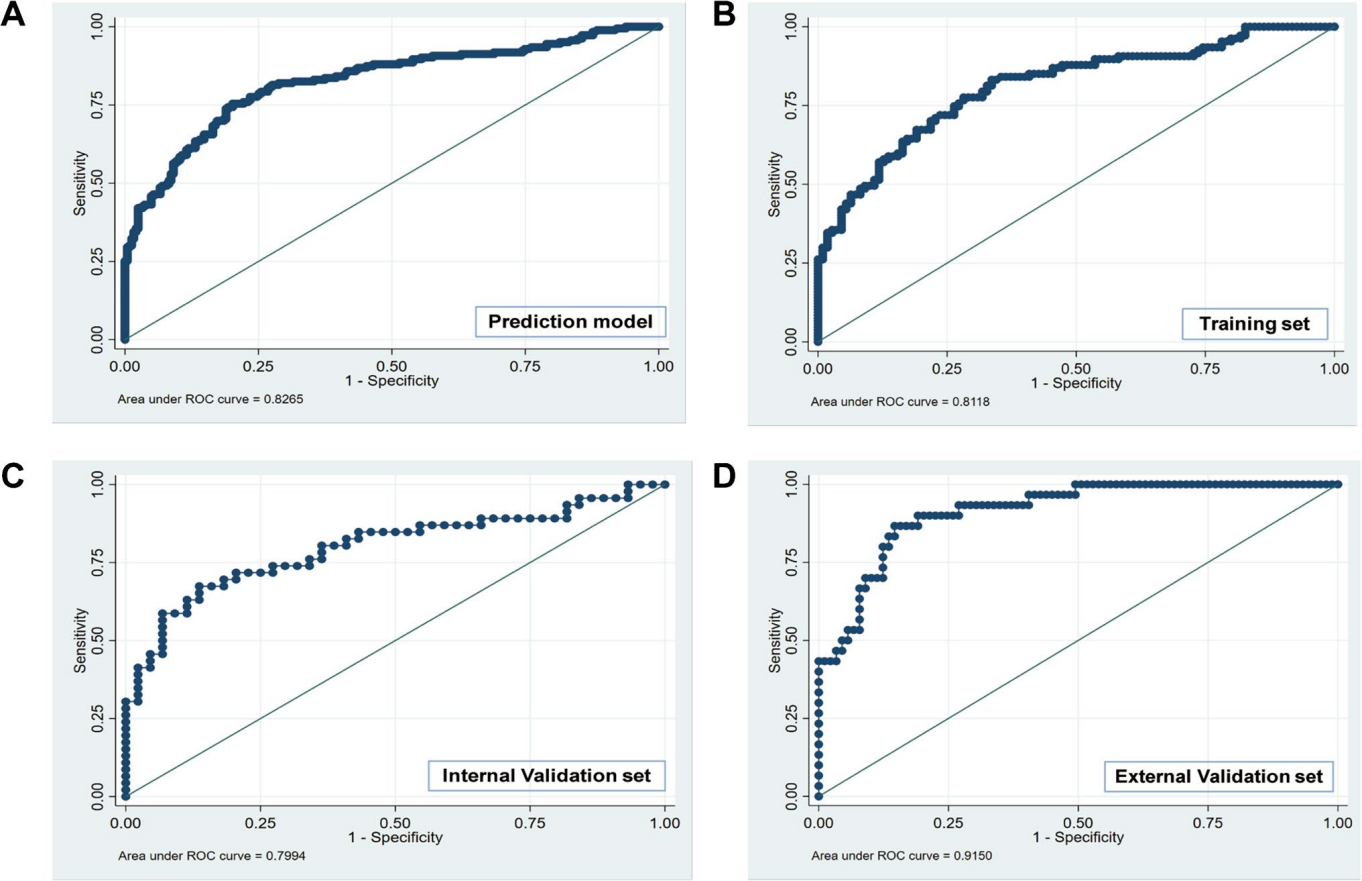
Receiver-operating characteristic curve and the corresponding area of the predictive model (indicators including upper respiratory infection, vomiting, PLT, WBC, and LC absolute counts). Note: **A** prediction model; **B** training set; **C** internal validation set; **D** external validation set

**Fig. 3:**
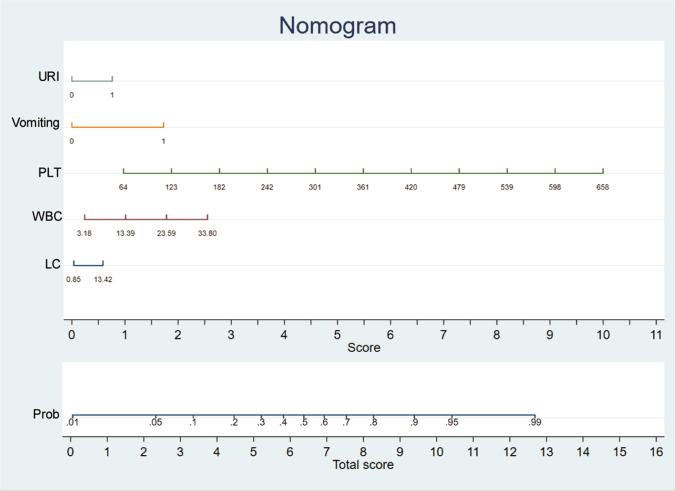
Nomogram predicting the risk assessment of NoV infection

According to the statistically significant results distinguishing NoV from non-NoV infection in univariate analysis, model 1 involved all univariate valid variables, while model 2 deleted the minimum combination of variables with the smallest AIC and the largest area under the ROC curve (AUC) to assure its best predictive performance (Table S2). Comparing the two prediction models, the AIC of model 2 was 237.76, which is lower than that of model 1 (249.99); model 2 was therefore selected as the prediction model. Subsequently, a nomogram was constructed to assess the possibility of NoV infection based on the multivariate logistic regression outputs for model 2. This diagnostic nomogram possessed good evaluation ability, as reflected by an AUC of 0.827 (95% *CI*: 0.785–0.868) (Fig. 2). Finally, the risk assessment prediction model is presented in the form of the nomogram (Fig. 3).

### Evaluation performance of prediction model in the training set, internal validation set, and external validation set

**Fig. 4:**
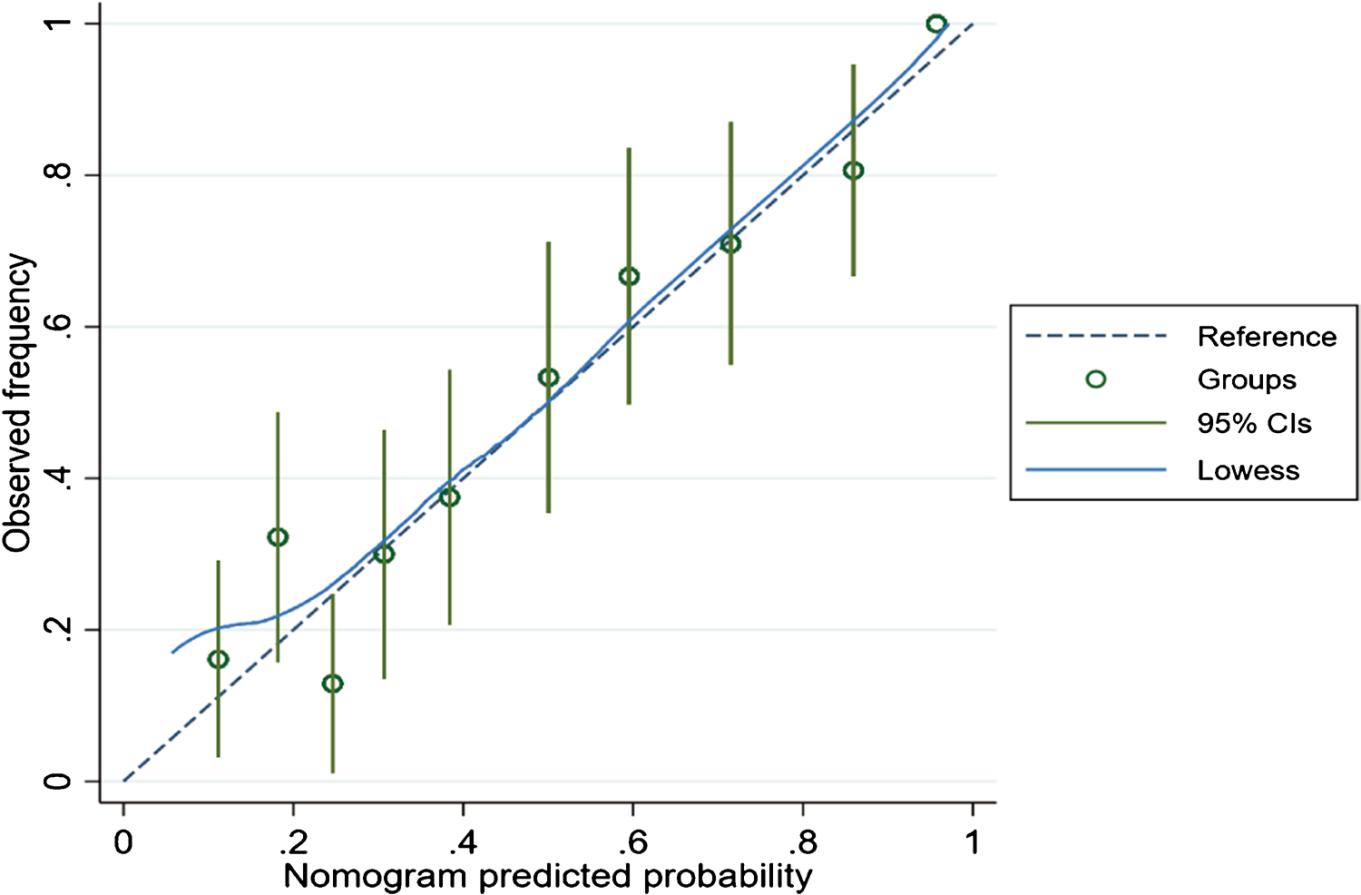
Calibration of the nomogram predicted system. Note: nomogram-predicted probability of NoV infection was plotted on the *x*-axis, actual diagnosis of NoV with qPCR results was plotted on the *y*-axis, and 95% *CI* was measured using Kaplan–Meier analysis. All predictions lie within the 10% margin of error (within the blue dots line)

**Fig. 5:**
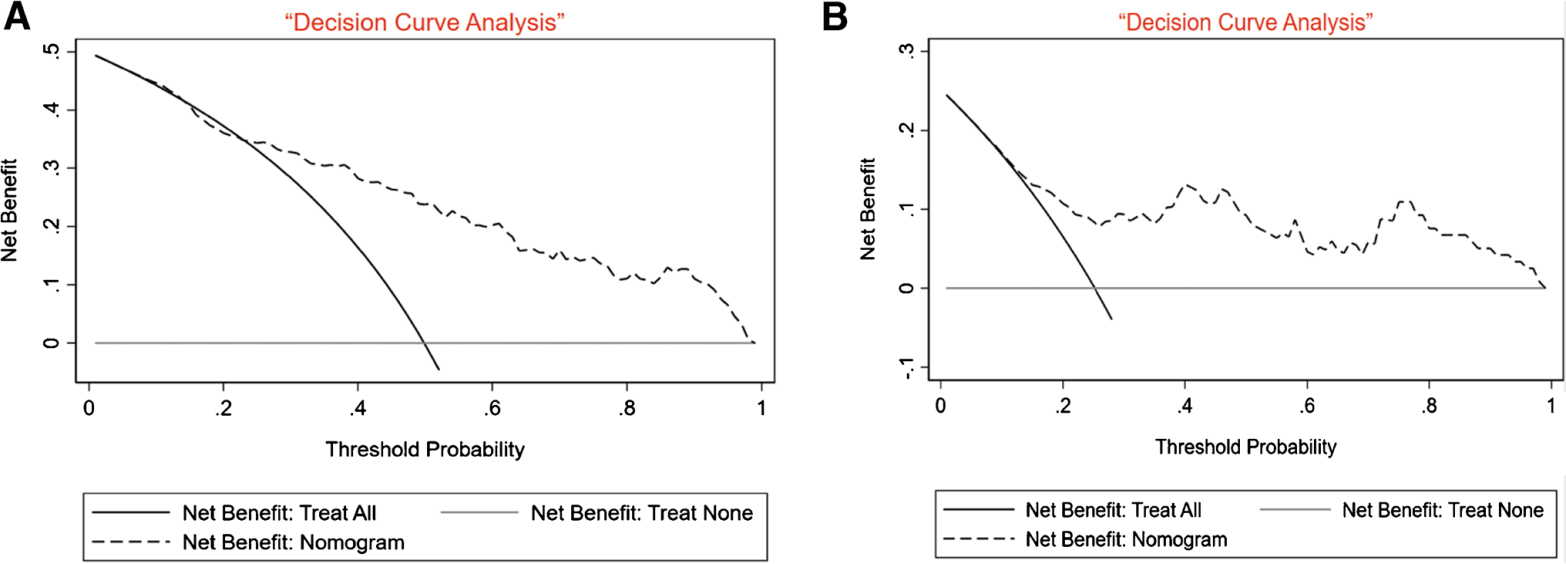
Decision curve analyses depicting the clinical net benefit of the prediction risk assessment of NoV infection nomogram. Note: **A** the decision curve analysis for NoV infection risk assessment nomogram in the internal validation set. **B** The decision curve analysis for NoV infection risk assessment nomogram in the external validation set. The *x*-axis shows the threshold probability. Threshold probability was defined as the minimum probability of disease at which further intervention would be warranted. The *y*-axis represents the net benefit, which is calculated across a range of threshold probabilities. Net benefit = sensitivity × prevalence-(1-specificity) × (1-prevalence) × w, where w is the odds at the threshold probability

All samples were randomly assigned to a training cohort (*n* = 217; 70%) and an internal validation cohort (*n* = 90, 30%). The prediction model presented a distinct ability to identify NoV infection, as reflected by an AUC of 0.812 (95% *CI*: 0.755–0.869) and 0.799 (95% *CI*: 0.705–0.894) in the training and internal validation sets, respectively (Fig. 2). Moreover, in the external validation set, this risk assessment model exhibited a more favorable discriminative power, as reflected by an AUC of 0.915 (95% *CI*: 0.862–0.968) (Fig. 2). This prediction model demonstrated good performance in the external validation set. Calibration of the nomogram-predicted system presented an ideal consistency result (Fig. 4). Finally, decision curve analysis (DCA) was used to assess the clinical utility of the diagnostic nomogram. Moreover, we applied DCAs to compare the performance of the models with respect to their clinical usefulness. These analyses revealed that the NoV infection risk assessment nomogram performed well (Fig. 5).

### Evaluation of the risk assessment prediction model for rapid differentiating between NoV and non-NoV infection

**Fig. 6:**
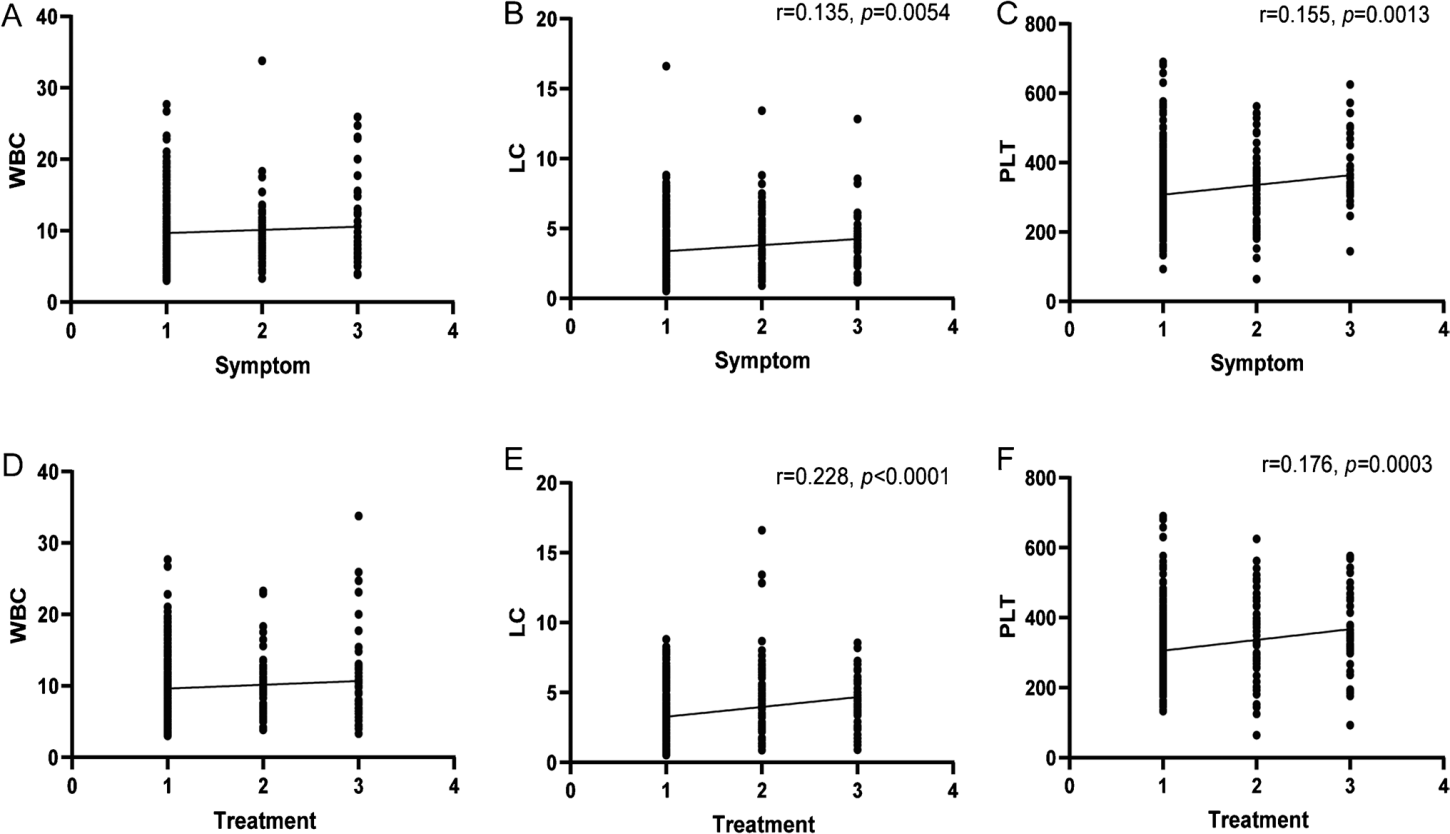
Correlation analysis of symptoms or treatments with significant clinical laboratory indicators of WBC counts, LC absolute counts, and PLT involved in risk assessment model of NoV infection nomogram

NoV infection has symptoms similar to those of diarrhea, abdominal pain, and vomiting, which are common in other acute gastroenteritis diseases. However, the spread of NoV infection causes far greater social damage than other gastrointestinal diseases. The successful construction of an accurate risk assessment prediction model for NoV infection is important to physicians. The significant variables entered into our NoV risk prediction model included common clinical symptoms and routine blood tests that can be easily acquired in routine clinical practice. The risk scoring system for NoV infection, that is, the nomogram, should be well applied in physician’s clinical practice (Fig. 3). By analyzing the correlation between significant laboratory indicators and different degrees of symptoms or treatments, it was found that WBC, LC, and PLT were positively correlated with the symptoms. In particular, the positive correlation coefficients between LC, PLT, and symptoms were *r* = 0.135 (*p* = 0.0054) and 0.155 (*p* = 0.0013), respectively, and the correlation coefficients between treatments were *r* = 0.228 (*p* < 0.0001) and 0.176 (*p* = 0.0003), as shown in Fig. 6.

In addition, we calculated the sensitivity, specificity, and concordance rate, as well as positive and negative predictive values of the prediction model. The results are shown in Table 4. We observed the concordance rate commonly applied in clinical practice. Therefore, we compared the concordance rate in different groups and found that it was 88.2% in the external validation set, which was the highest rate, 76.5% in the prediction model, 73.3% in the training set, and 77.8% in the internal validation set. In addition, a nomogram was constructed based on the aforementioned indicators to facilitate rapid individualized risk assessment of NoV infection by physicians.

**Table 4:** Evaluation of different model with clinical diagnostic indicators

Model	Sensitivity (%)	Specificity (%)	Concordance rate (%)	Positive predictive value (%)	Negative predictive value (%)
Prediction model	64.5	85.6	76.5	77.1	76.2
Training set	72	74.5	73.3	73.3	73.2
Internal validation set	69.6	86.4	77.8	77.1	84.2
External validation set	66.7	95.5	88.2	88.3	89.5

## Discussion

In the present study, we analyzed the indicators of routine blood examination and clinical symptoms to assess the risk of NoV infection and improve its diagnostic efficiency. A risk assessment model for NoV infection diagnosis was established in patients undergoing routine blood examinations and manifesting basic clinical symptoms. This prediction model incorporated five valid items: PLT, WBC, LC, URI, and vomiting. Furthermore, the model had better predictive ability in the external than in the internal validation set. We then compared the sensitivity, specificity, concordance rate, and positive and negative predictive values of our prediction model; the values mentioned above in the external validation set showed better performance. Therefore, the nomogram prediction model may be considered useful for the differential diagnosis of NoV in routine clinical practice.

Although successful in vitro culture of NoV has been reported, it is only limited to special intestinal epithelial cells or B cells, and the high technical requirements of culture are generally difficult to achieve [17, 18]. Moreover, cell culture rarely isolated growth and is relatively slow compared to qPCR, although with extraction and qPCR techniques, those 5 h diagnostics can be reduced to even 2 h. Since NoV is rarely isolated and cultured, real-time qPCR is currently the gold standard for diagnosing NoV infection, despite its methodological limitations [3, 19–21]. By comparing the models in different sets, its specificity of above 85% was higher than the sensitivity of 70%, implying a lower chance of misdiagnosis and a better positive predictive value overall. Although the sensitivity of our different model sets was only approximately 70%, the negative predictive value exceeded 80%. We may need to incorporate more sensitive assays to improve the sensitivity of the prediction model. Diagnostic accuracy is the focus of clinician attention, and the concordance rate in different model sets was almost 80%.

Our risk assessment model for predicting NoV infection was evaluated in terms of the AUC, which is presented as an intuitive nomogram [16, 22]. By comparing the size of the AUC for the different models, we found that our model presented the best predictive performance for external validation. The AUC value of 0.915 (95% *CI*: 0.862–0.968) was significantly higher than that of the prediction model and the internal validation set. Moreover, it suggests that the predictive model we constructed for the risk assessment of NoV infection has a better clinical predictive performance. The risk prediction model was constructed using multivariate logistic regression analysis and incorporated routine laboratory blood indicators as well as common symptoms and clinical signs [23, 24]. We then correlated the valid routine blood indicators in the model with different degrees of symptoms and clinical treatments. We found that leukocytes, lymphocytes, and platelets were significantly positively correlated with clinical symptoms and treatments. In particular, the positive correlation between lymphocytes, platelets, and both clinical symptoms and treatment suggested that higher lymphocyte and platelet counts indicate more gastrointestinal clinical symptoms and the need for more intensive clinical management. When NoV infection is commonly combined with acute upper respiratory tract infection, there is a significant increase in leukocyte and neutrophil counts; therefore, prophylactic treatment with antibiotics is required except in cases of a viral infection. The symptoms of NoV infection are variable and include diarrhea, nausea, vomiting, and other gastrointestinal malaise [8, 9]. In our model, a relationship was found between vomiting, upper respiratory infections, and NoV infections due to NoV infections occurring in the autumn and winter seasons and co-infection with upper respiratory infections. Vomiting is the main symptom of NoV infection in children, which is significantly different from diarrheal symptoms that are the main complaint of NoV infection in adults.

We constructed a prediction model to support the risk assessment of NoV infections in physicians’ clinical trials. We focused on selected laboratory indicators, such as routine blood tests, as these indicators are regularly tested in almost every visiting patient and thus can be easily implemented in clinical practice. In terms of the laboratory blood routine results, we found that the total number of WBC, LC, and NC in the NoV qPCR-positive group was obviously higher than those in the negative group, indicating that the immune cells were activated in the body after viral infection. Viral infections are prone to reactive increases in lymphocytes, but neutrophils and total leukocytes are not elevated and may only be elevated in combination with acute upper respiratory infections. This minimal change activated the RBC system after the NoV infection. According to platelet count and related indicators, PLT was significantly higher in the NoV infection than in the non-infection group. This profile is relatively different from SARS-CoV-2, but similar to HCV, HIV infection, and other gastrointestinal virus infections, such as enterovirus [23–28]. It has been reported that PLT was significantly increased in children with EV71 infection, which serve as immune regulatory cells that affect the pathogenesis of virus infection. PLT and PCT levels are positively associated with the severity of EV71 [29].

Despite these strengths, there are some limitations that should be considered when interpreting the results of our study. First, potential selection bias might have occurred because of the retrospective nature of the study. Second, although our model for risk assessment to predict NoV infection has good specificity and can accurately diagnose people with NoV infection, its sensitivity requires further improvement. Subsequently, we can identify more sensitive tests to be incorporated into the model to improve its sensitivity. Finally, the model only considered partial clinical indicators and factors. Future studies may consider incorporating the consistency of the stool, other potential clinical risk factors, and other high-throughput quantitative technologies of viral loads to improve the prediction performance.

## Conclusion

We constructed a risk assessment model for the differential diagnosis of NoV infection. It contained five valid variables, including PLT, WBC, LC, URI, and vomiting. These indicators are readily available, and the external validation set achieved better predictive efficacy, which is consistent with the gold standard. In conclusion, elevated levels of PLT, WBC, LC, URI, and vomiting are risk factors for the occurrence of NoV infection in patients with acute gastroenteritis. These indicators of routine blood examination and accompanying symptoms are easily obtained; thus, this risk assessment model can assist in the differential diagnosis of NoV infection in daily clinical practice. In future studies, we will probably use a scoring system to screen severe cases in immunocompromised individuals.

## Supplementary Information

Below is the link to the electronic supplementary material.

Supplementary file1 (DOCX 22 KB)

## Data Availability

The datasets generated during and/or analyzed during the current study are available in the [prediction model raw data] repository, [https://kdocs.cn/l/cugVRAkTpOpg] in this study is available from the corresponding author on reasonable request.
