# Supplementary material for: Development and validation of an efficient nomogram for risk assessment of norovirus infection in pediatric patients
Source: Eur J Clin Microbiol Infect Dis. 2022 Oct 25;41(12):1433–43. doi: 10.1007/s10096-022-04510-8 (PMC9592877; doi:10.1007/s10096-022-04510-8)
Supplement: Supplementary file 1 — Supplementary file1 (DOCX 22 KB) [file 10096_2022_4510_MOESM1_ESM.docx]

**Development and validation of an efficient nomogram for risk assessment of norovirus infection in pediatric patients**

Taojun He^1#^, Xiaohua Chen^2#^, Yilin Deng^1^, Bin Li^1^, Hongmei Wang^3^, Qinjin Wang^1^, Aixia Zhai^1^, Liang Shi^1^, Ying Chen^1^*, Chao Wu^1^*

1. Department of Laboratory Medicine, The Eighth Affiliated Hospital of Sun Yat-sen University, Shenzhen 518033, Guangdong Province, China;
2. Department of Digestive Endoscopy Center, The Eighth Affiliated Hospital of Sun Yat-sen University, Shenzhen 518033, Guangdong Province, China;
3. Department of Infectious Diseases, Shenzhen Children's Hospital, Shenzhen 518000, Guangdong Province, China.

Corresponding authors: Chao Wu: [wuchao99261@163.com](mailto:wuchao99261@163.com)

Ying Chen: [cheny883@mail.sysu.edu.cn](mailto:cheny883@mail.sysu.edu.cn)

Table S1

Table S2

Table S1. Demographics features of study population

| Group | NoV qPCR negative（N = 154/89） | NoV qPCR positive（N = 153/30） | χ^2^ value / t value | *p*-value |
| --- | --- | --- | --- | --- |
| **Internal set**  sex |  |  | 2.111 | 0.160 |
| male | 101 | 88 |  |  |
| female | 53 | 65 |  |  |
| age(y) | 2.82 ± 2.49 | 2.34 ± 2.72 | 1.601 | 0.110 |
| **External set** |  |  |  |  |
| sex |  |  | 0.002 | 1.000 |
| male | 56 | 19 |  |  |
| female | 33 | 11 |  |  |
| age(y) | 2.65 ± 2.04 | 2.19 ± 1.60 | 1.116 | 0.267 |

Table S2-A. Coefficient of multiple logistic regression in model 1

| Model 1 | Odds Ratio | *p* value | *95% Confidence interval* | |
| --- | --- | --- | --- | --- |
| WBC | 1.055 | 0.195 | 0.973 | 1.144 |
| LC | 1.047 | 0.675 | 0.843 | 1.300 |
| EO | 1.392 | 0.697 | 0.263 | 7.369 |
| MCV | 0.986 | 0.681 | 0.921 | 1.055 |
| RDW-CV | 0.955 | 0.671 | 0.772 | 1.181 |
| PLT | 1.011 | **<0.001** | 1.007 | 1.016 |
| Vomiting | 4.250 | **0.003** | 1.657 | 10.902 |
| URI | 2.002 | 0.062 | 0.966 | 4.151 |
| Community related | 0.961 | 0.969 | 0.129 | 7.171 |
| Diarrhea | 1.411 | 0.556 | 0.448 | 4.450 |
| Cough | 0.598 | 0.479 | 0.144 | 2.481 |
| Convulsion | 0.845 | 0.828 | 0.184 | 3.869 |

Table S2-B. Coefficient of multiple logistic regression in model 2

| Model 2 | Odds Ratio | *p* value | *95% Confidence interval* | |
| --- | --- | --- | --- | --- |
| WBC | 1.057 | 0.178 | 0.975 | 1.144 |
| LC | 1.032 | 0.744 | 0.853 | 1.249 |
| PLT | 1.011 | **<0.001** | 1.007 | 1.015 |
| Vomiting | 3.514 | **0.003** | 1.534 | 8.051 |
| URI | 1.740 | 0.110 | 0.882 | 3.434 |
